# Ensemble machine learning prediction of hyperuricemia based on a prospective health checkup population

**DOI:** 10.3389/fphys.2024.1357404

**Published:** 2024-04-11

**Authors:** Yongsheng Zhang, Li Zhang, Haoyue Lv, Guang Zhang

**Affiliations:** ^1^ Health Management Center, The First Affiliated Hospital of Shandong First Medical University and Shandong Provincial Qianfoshan Hospital, Jinan, China; ^2^ Institute of Health Management, The First Affiliated Hospital of Shandong First Medical University and Shandong Provincial Qianfoshan Hospital, Jinan, China; ^3^ Shandong Engineering Laboratory of Health Management, The First Affiliated Hospital of Shandong First Medical University and Shandong Provincial Qianfoshan Hospital, Jinan, China; ^4^ Department of Pharmacology, Jinan Central Hospital Affiliated to Shandong First Medical University, Jinan, China

**Keywords:** hyperuricemia, prediction model, machine learning, stacking ensemble, risk factors

## Abstract

**Objectives:** An accurate prediction model for hyperuricemia (HUA) in adults remain unavailable. This study aimed to develop a stacking ensemble prediction model for HUA to identify high-risk groups and explore risk factors.

**Methods:** A prospective health checkup cohort of 40899 subjects was examined and randomly divided into the training and validation sets with the ratio of 7:3. LASSO regression was employed to screen out important features and then the ROSE sampling was used to handle the imbalanced classes. An ensemble model using stacking strategy was constructed based on three individual models, including support vector machine, decision tree C5.0, and eXtreme gradient boosting. Model validations were conducted using the area under the receiver operating characteristic curve (AUC) and the calibration curve, as well as metrics including accuracy, sensitivity, specificity, positive predictive value, negative predictive value, and F1 score. A model agnostic instance level variable attributions technique (iBreakdown) was used to illustrate the black-box nature of our ensemble model, and to identify contributing risk factors.

**Results:** Fifteen important features were screened out of 23 clinical variables. Our stacking ensemble model with an AUC of 0.854, outperformed the other three models, support vector machine, decision tree C5.0, and eXtreme gradient boosting with AUCs of 0.848, 0.851 and 0.849 respectively. Calibration accuracy as well as other metrics including accuracy, specificity, negative predictive value, and F1 score were also proved our ensemble model’s superiority. The contributing risk factors were estimated using six randomly selected subjects, which showed that being female and relatively younger, together with having higher baseline uric acid, body mass index, γ-glutamyl transpeptidase, total protein, triglycerides, creatinine, and fasting blood glucose can increase the risk of HUA. To further validate our model’s applicability in the health checkup population, we used another cohort of 8559 subjects that also showed our ensemble prediction model had favorable performances with an AUC of 0.846.

**Conclusion:** In this study, the stacking ensemble prediction model for HUA was developed, and it outperformed three individual models that compose it (support vector machine, decision tree C5.0, and eXtreme gradient boosting). The contributing risk factors were identified with insightful ideas.

## Introduction

Hyperuricemia (HUA) is a disease characterized by elevated blood uric acid due to disorders of purine metabolism and/or impaired uric acid excretion in the body. In recent years, the prevalence and disease burden of HUA have gradually increased globally (Dehlin et al., 2020), and a cross-sectional study shows that the overall prevalence of HUA in China has increased from 11.1% to 14.0% within 3 years, which demonstrates a significant ascending trend (Zhang et al., 2021). Many studies indicate that HUA often develops into gout and is closely related to the development of cardiovascular diseases, hypertension, obesity and other diseases (Maloberti et al., 2020; McCormick et al., 2022; Han et al., 2023; Lin et al., 2024), which has become a serious public health problem.

Machine learning is a type of artificial intelligence that enables computer to automatically extract useful information from large amounts of data and make intelligent decisions and predictions. Ensemble learning is one of the machine learning strategies that aggregate the power of multiple models to enhance prediction. There are three main types of ensemble learning algorithms: bagging, boosting, and stacking, each with its unique way of model combination (Zhou, 2021). Stacking trains multiple first-level models with different algorithms on the same dataset and combines their predictions using a second-level model, known as the meta-learner, to produce one more accurate and robust prediction (Mahajan et al., 2023). We aimed to use the stacking ensemble technique to build an accurate HUA risk prediction model, integrating the results of support vector machine (SVM), decision tree C5.0 (C5.0), and eXtreme gradient boosting (XGBoost) to improve the final performance.

Thus far, various studies worldwide have identified different risk factors associated with the occurrence of HUA, such as age, gender, waist circumference, drinking, smoking, obesity, hypertension, dyslipidemia and triglyceride-glucose index (Dong et al., 2022; Piao et al., 2022; Wang et al., 2022; Ding et al., 2023; Lyu et al., 2023; Teramura et al., 2023; Liu et al., 2024). Moreover, several prediction models for HUA have been developed using machine learning algorithms (Lee et al., 2019; Zeng et al., 2020; Gao et al., 2021; Huang et al., 2022; Zhu et al., 2023). However, these models were either tailored for specific subgroup or did not incorporate sufficient predictors. Additionally, none of them attempted the ensemble approach, resulting in poor predictive performance and a lack of practical application. Therefore, it is very necessary to develop a more accurate prediction model for the risk of HUA using the ensemble strategy and develop an easy-to-use risk calculator for clinical settings.

In the following sections, we initiate with an overview of the research methodology, encompassing the study population, data preprocessing, and all the statistical methods. Then, we present a statistical description of the study population, detailing the feature selection, model construction, and evaluation processes, unveiling the black box our model, and building a risk calculator. At last, we engage in an extensive discussion highlighting the superiority of our methods, comparing our model with existing ones, and delving into the risk factors.

## Materials and methods

### Study design and participants

This study was a prospective cohort study based on a large longitudinal health checkup cohort in the First Affiliated Hospital of Shandong First Medical University and was approved by the Ethics Committee of this hospital. Subjects without HUA at their first checkup in the year 2021 and without any missing variables were enrolled. All subjects were followed up for 1 year, and their HUA status were checked at the end of follow up in the year of 2022.

### Data collection and preprocessing

By reviewing previous studies, we identified 23 variables from routine health checkup data that are possibly associated with HUA. They were age, gender, body mass index (BMI), systolic blood pressure (SBP), diastolic blood pressure (DBP), alanine aminotransferase (ALT), aspartate aminotransferase (AST), γ-glutamyl transpeptidase (GGT), total bilirubin (TBil), total protein (TP), albumin (Alb), blood urea nitrogen (BUN), creatinine (Cr), estimated glomerular filtration rate (EGFR), triglycerides (TG), total cholesterol (TC), high-density lipoprotein cholesterol (HDL), low-density lipoprotein cholesterol (LDL), fasting blood glucose (FBG), white blood cell count (WBC), neutrophil count (NEUT), baseline uric acid (BUA) and the fatty liver status. BMI was determined as dividing the weight (kg) by the square of the height (m^2^). SBP and DBP were measured on the right upper arm after the subjects seated for a 5-min rest. After a 12-h fasting period, peripheral blood samples were collected in the morning to measure the following blood variables: ALT, AST, GGT, TBil, TP, Alb, BUN, Cr, EGFR, TG, TC, HDL, LDL, FBG, WBC, NEUT and BUA. All laboratory tests were performed following standard protocols at the Department of Laboratory. Fatty liver status was diagnosed by certified imaging physicians through abdominal ultrasound examination. The diagnostic threshold for HUA was established as serum uric acid level of 420 μmol/L for males and 360 μmol/L for females (Endocrinology, 2020).

### Statistical analysis

Descriptive analysis for the baseline characteristics was performed. Statistical significance for quantitative data was evaluated using Student’s t-test or nonparametric Wilcoxon test, and the Chi-square test was employed for the qualitative data.

**FIGURE 1:**
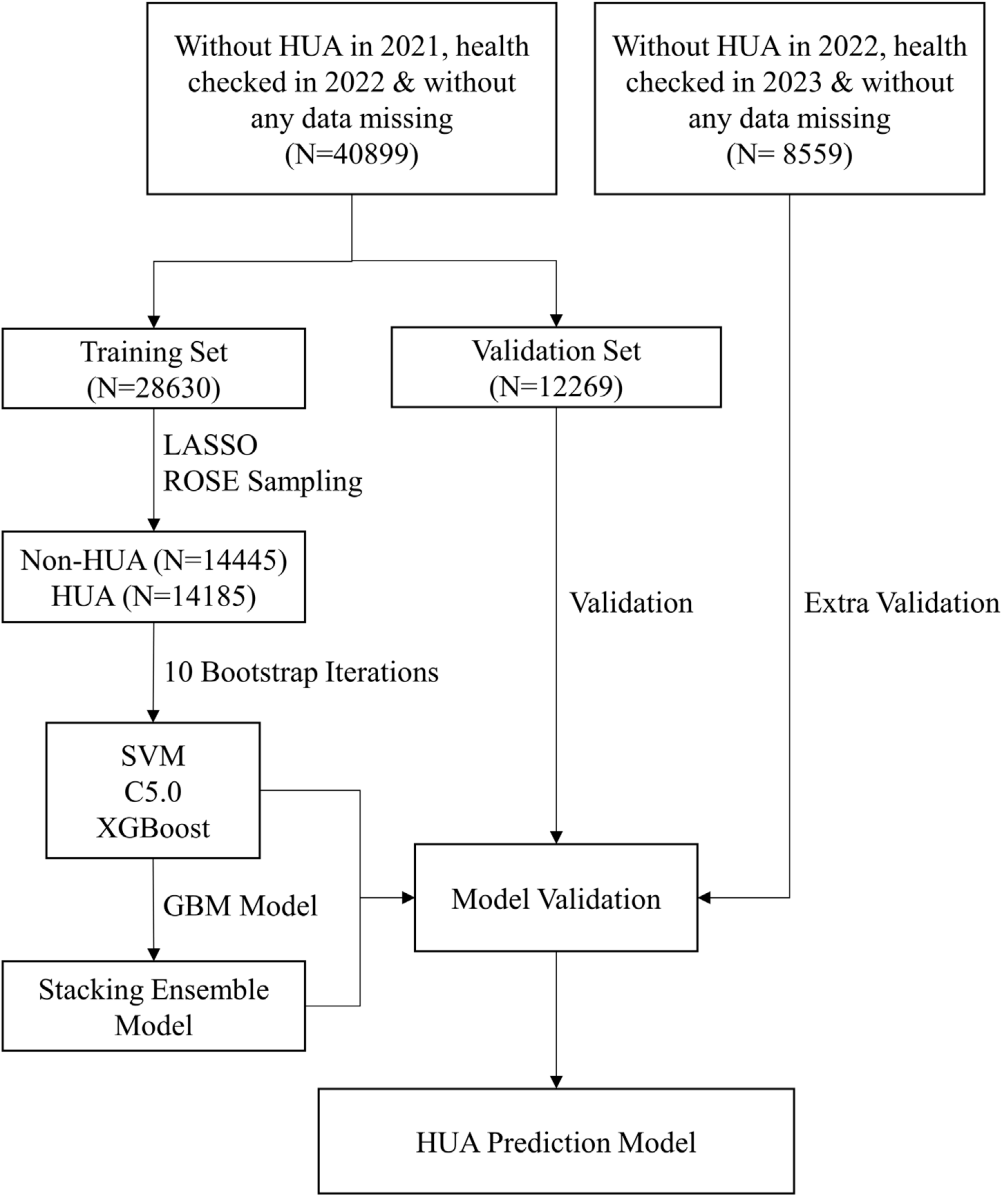
The flowchart of our ensemble prediction model. Abbreviations: HUA, hyperuricemia; SVM, support vector machine; C5.0, decision tree C5.0; XGBoost, eXtreme gradient boosting; GBM, gradient boosting machine model.

Prediction model was constructed and evaluated, as shown in Figure 1. Firstly, the final dataset was randomly divided into the training set, comprising 70% of the subjects, and the validation set, comprising the remaining 30% (Lyu et al., 2020; Chen et al., 2021). Then, we utilized LASSO regression for feature selection (Friedman et al., 2010; Sauerbrei et al., 2011), and screened 15 important features among the 23 clinical variables by adding a penalty function. Next, to handle the disparity in the frequencies of the observed classes and generate a steady prediction model, the ROSE sampling from the R ***ROSE*** package was used (Nicola et al., 2014), which down-sampled the majority class and synthesized new data in the minority class. Then, our models were trained using the platform provided by the R ***caretEnsemble*** package. The SVM, C5.0, XGBoost, and the stacking ensemble model assembling these three models were developed based on the training set using 15 selected features. Then, we conducted internal validation of our models using the validation set and obtained estimates of the area under the receiver operating characteristic curve (AUC) as well as multiple metrics for evaluating the performance of our models, including accuracy, sensitivity, specificity, positive predictive value (PPV), negative predictive value (NPV) and F1 score. At the same time, the calibration curve of each model was depicted. All of the above evaluations were employed to assess the discrimination of our models, which refers to their ability to effectively distinguish between individuals who had high risks of diseases and those who did not. Furthermore, a model agnostic instance level variable attributions technique (iBreakdown) was used to illustrate the black-box nature of our ensemble model (Gosiewska and Biecek, 2019), and contributing risk factors were identified. Lastly, we developed a dynamic risk calculator based on the R ***shiny*** package for ease of clinical use, and further estimated its validity using decision curve analysis.

All statistical tests were two-sided with a type I error of 0.05, and *p*-value <0.05 were considered statistically significant. Statistical analysis was carried out using software R version 4.2.2 and Python version 3.10.8.

## Results

### Baseline characteristics

For the health checkup cohort of 40899 subjects, the mean (SD) ages for males and females were 47.4 (14.0) and 45.4 (13.6) years old, respectively. At the end of the follow-up period, 4055 HUA cases (2770 males and 1285 females) were diagnosed, resulting in an incidence rate of 99.15/1000 person-years. The baseline characteristics of 36844 non-HUA subjects and 4055 HUA subjects were listed below, as shown in Table 1.

**TABLE 1:** Baseline characteristics of subjects in different groups.

	Non-hyperuricemia (N = 36844)	Hyperuricemia (N = 4055)	*p*-value
**Categorical variables**			
**Gender**			
Female	17088 (46.4%)	1285 (31.7%)	<0.001
Male	19756 (53.6%)	2770 (68.3%)	
**Fatty_liver**			
Non-Fatty_liver	22102 (60.0%)	1435 (35.4%)	<0.001
Fatty_liver	14742 (40.0%)	2620 (64.6%)	
**Continuous variables**			
**Age**	47.394 (14.012)	45.401 (13.639)	<0.001
**BMI**	24.074 (3.443)	25.985 (3.501)	<0.001
**SBP**	125.715 (17.723)	129.029 (16.832)	<0.001
**DBP**	76.457 (11.287)	79.446 (11.318)	<0.001
**ALT**	19.112 (23.925)	25.911 (21.191)	<0.001
**AST**	18.794 (11.349)	21.229 (9.562)	<0.001
**GGT**	24.139 (23.247)	35.120 (33.895)	<0.001
**TBil**	12.245 (5.587)	12.915 (5.874)	<0.001
**TP**	73.573 (3.974)	74.587 (3.908)	<0.001
**Alb**	47.144 (2.630)	47.797 (2.676)	<0.001
**BUN**	4.715 (1.263)	5.025 (1.182)	<0.001
**Cr**	70.909 (16.018)	76.941 (13.384)	<0.001
**EGFR**	105.755 (15.546)	103.066 (15.143)	<0.001
**TG**	1.296 (0.802)	1.715 (1.061)	<0.001
**TC**	4.776 (0.922)	4.990 (0.939)	<0.001
**HDL**	1.370 (0.303)	1.280 (0.270)	<0.001
**LDL**	2.685 (0.703)	2.856 (0.731)	<0.001
**FBG**	5.110 (1.25)	5.122 (1.07)	0.481
**WBC**	6.206 (1.538)	6.646 (1.525)	<0.001
**NEUT**	3.434 (1.134)	3.652 (1.136)	<0.001
**BUA**	296.630 (64.781)	362.269 (52.317)	<0.001

Note: The names of all the variable are shown in bold.

Abbreviations: BMI, body mass index; SBP, systolic blood pressure; DBP, diastolic blood pressure; ALT, alanine aminotransferase; AST, aspartate aminotransferase; GGT, γ-glutamyl transpeptidase; TBil, total bilirubin; TP, total protein; Alb, albumin; BUN, blood urea nitrogen; Cr, creatinine; EGFR, estimated glomerular filtration rate; TG, triglycerides; TC, total cholesterol; HDL, high-density lipoprotein cholesterol; LDL, low-density lipoprotein cholesterol; FBG, fasting blood glucose; WBC, white blood cell count; NEUT, neutrophil count; BUA, baseline uric acid.

### Feature selection

**FIGURE 2:**
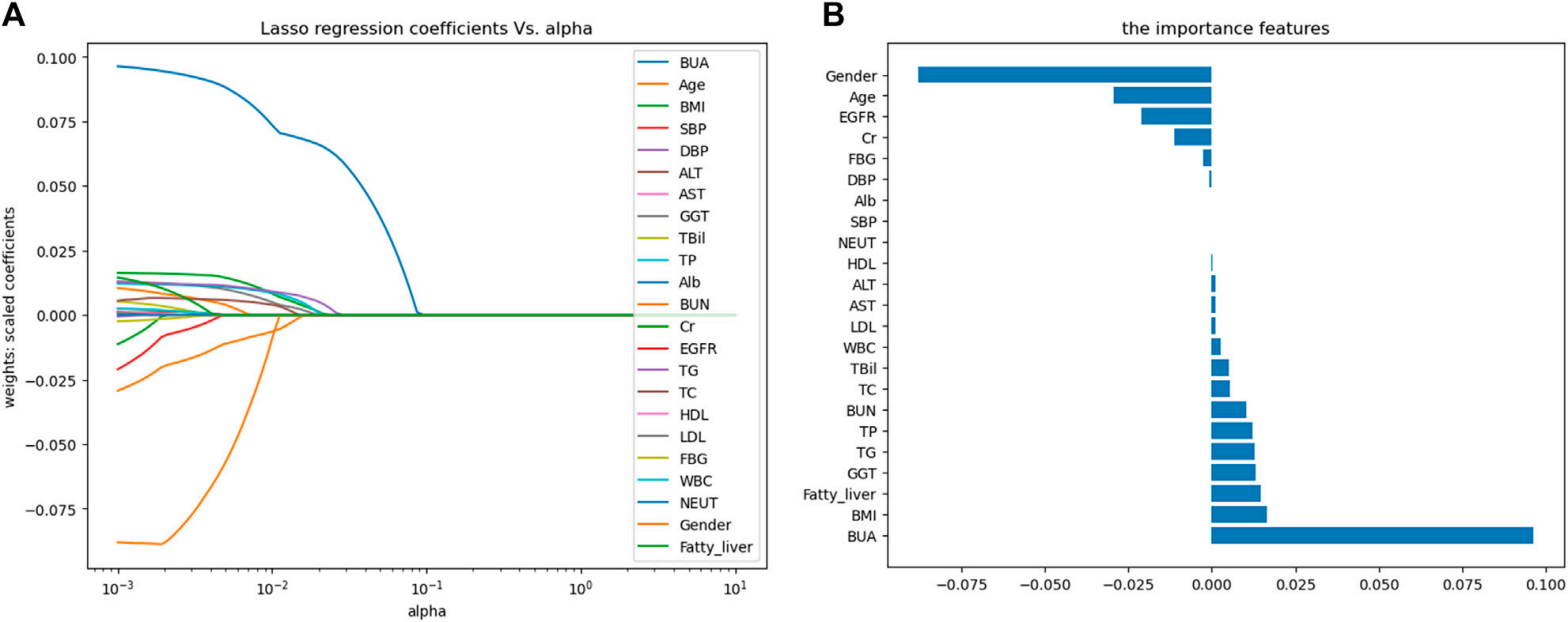
Variable selection based on LASSO regression. **(A)** LASSO coefficient path map; **(B)** Feature importance map. Abbreviations: BMI, body mass index; SBP, systolic blood pressure; DBP, diastolic blood pressure; ALT, alanine aminotransferase; AST, aspartate aminotransferase; GGT, γ-glutamyl transpeptidase; TBil, total bilirubin; TP, total protein; Alb, albumin; BUN, blood urea nitrogen; Cr, creatinine; EGFR, estimated glomerular filtration rate; TG, triglycerides; TC, total cholesterol; HDL, high-density lipoprotein cholesterol; LDL, low-density lipoprotein cholesterol; FBG, fasting blood glucose; WBC, white blood cell count; NEUT, neutrophil count; BUA, baseline uric acid.

Predicting features were filtered by LASSO regression, and 15 features were finally screened out of 23 variables, including age, gender, BMI, GGT, TBil, TP, BUN, Cr, EGFR, TG, TC, FBG, WBC, BUA and the fatty liver status, as shown in Figure 2. The figure on the left was the LASSO coefficient path diagram, where each curve represents the trajectory of the coefficient of each variable, and the variables first reached to point 0 were excluded. The figure on the right is the feature importance diagram, which shows how much every feature is related to the outcome by ranking their coefficients.

### Construction of prediction models

**FIGURE 3:**
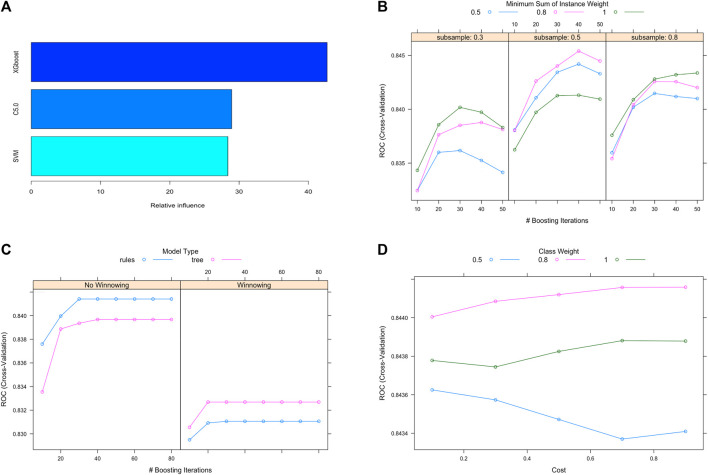
Ensemble model construction and hyperparameter tuning. **(A)** Contributions of individual models in the stacking ensemble model. **(B)**, **(C)**, **(D)** Hyperparameter tuning process for the XGBoost, C5.0 and SVM models. Abbreviations: XGBoost, eXtreme gradient boosting; C5.0, decision tree C5.0; SVM, support vector machine.

First of all, 14445 non-HUA subjects and 14185 HUA subjects were generated from the training set using the ROSE sampling method. 10 bootstrapped datasets from the training set were used to train three individual machine learning models, SVM, C5.0, and XGBoost. The grid search strategy was used for hyperparameters selection. Then, the gradient boosting machine model was applied as the meta learner to stack these three individual models together into our ensemble model. We can see that the XGBoost takes the largest proportion of influence in our ensemble model, as shown in Figure 3A. The hyperparameter tuning process of the component models, XGBoost, C5.0, and SVM are shown in Figures 3B–D respectively. The area under the receiver operating characteristic curve (ROC) showed increasing trends with boosting iterations.

**FIGURE 4:**
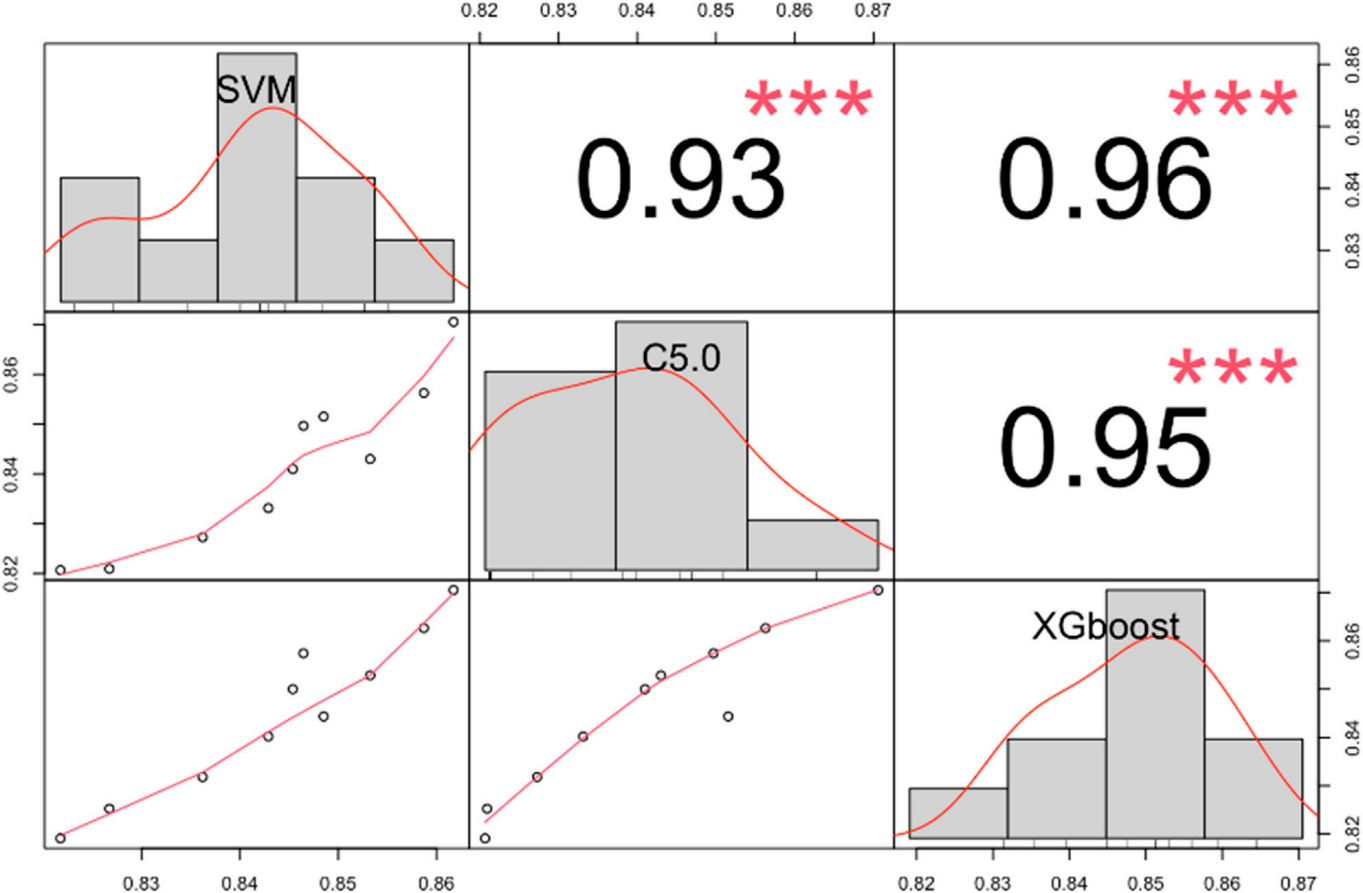
The correlation matrix shows the AUC for SVM, C5.0, and XGBoost models in different bootstrapped datasets. ***, *p* < 0.001. Abbreviations: AUC, the area under the receiver operating characteristic curve; SVM, support vector machine; C5.0, decision tree C5.0; XGBoost, eXtreme gradient boosting.

The AUC for each of the 10 bootstrapped datasets were obtained, as depicted in Figure 4, and they varied across different subsets for the three machine learning models. Also, the correlations between each pair of models were examined, and they showed significant statistical differences, which indicated that each model captured distinct aspects of the data. In this case, there is a good chance that our ensemble model can enhance predictive performance even further while stacking these three machine learning models together.

### Evaluation of prediction models

**FIGURE 5:**
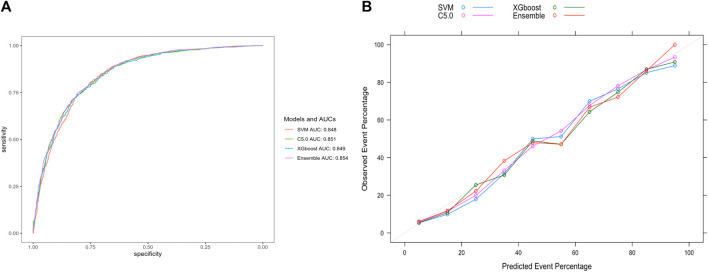
Evaluation of model performance on the validation set. **(A)** ROC curve determines which model has better classification ability. **(B)** Calibration curve shows the consistency between observed and predicted probabilities. Abbreviations: ROC, the receiver operating characteristic curve; AUC, the area under the receiver operating characteristic curve; XGBoost, eXtreme gradient boosting; C5.0, decision tree C5.0; SVM, support vector machine; Ensemble, stacking ensemble model.

For ease of comparison, the ROC curves of four models on the validation set were depicted in a single plot, as shown in Figure 5A. The stacking ensemble model with an AUC of 0.854, outperformed the other three models, SVM, C5.0, and the XGBoost with AUCs of 0.848, 0.851 and 0.849, respectively. Moreover, the ensemble model outperformed the other three models in terms of calibration accuracy with fewer deviations from the diagonal, as shown in Figure 5B. Other metrics for evaluating our models, including accuracy, sensitivity, specificity, PPV, NPV, and F1 score were also presented, which further proved the ensemble model’s superiority over the other three models, as shown in Table 2.

**TABLE 2:** Other performance metrics of different models on the validation set.

Model	Accuracy	Sensitivity	Specificity	Positive predictive value	Negative predictive value	F1 score
support vector machine **(SVM)**	0.923	0.813	0.934	0.554	0.980	0.659
decision tree C5.0 (**C5.0)**	0.928	0.830	**0.938**	0.575	0.982	0.680
eXtreme gradient boosting **(XGBoost)**	0.925	0.871	0.930	0.556	0.986	0.679
stacking ensemblemodel **(Ensemble)**	**0.931**	**0.877**	0.936	**0.581**	**0.987**	**0.699**

Note: The values of the best performing models for each metric are shown in bold.

### Ensemble model interpretation

**FIGURE 6:**
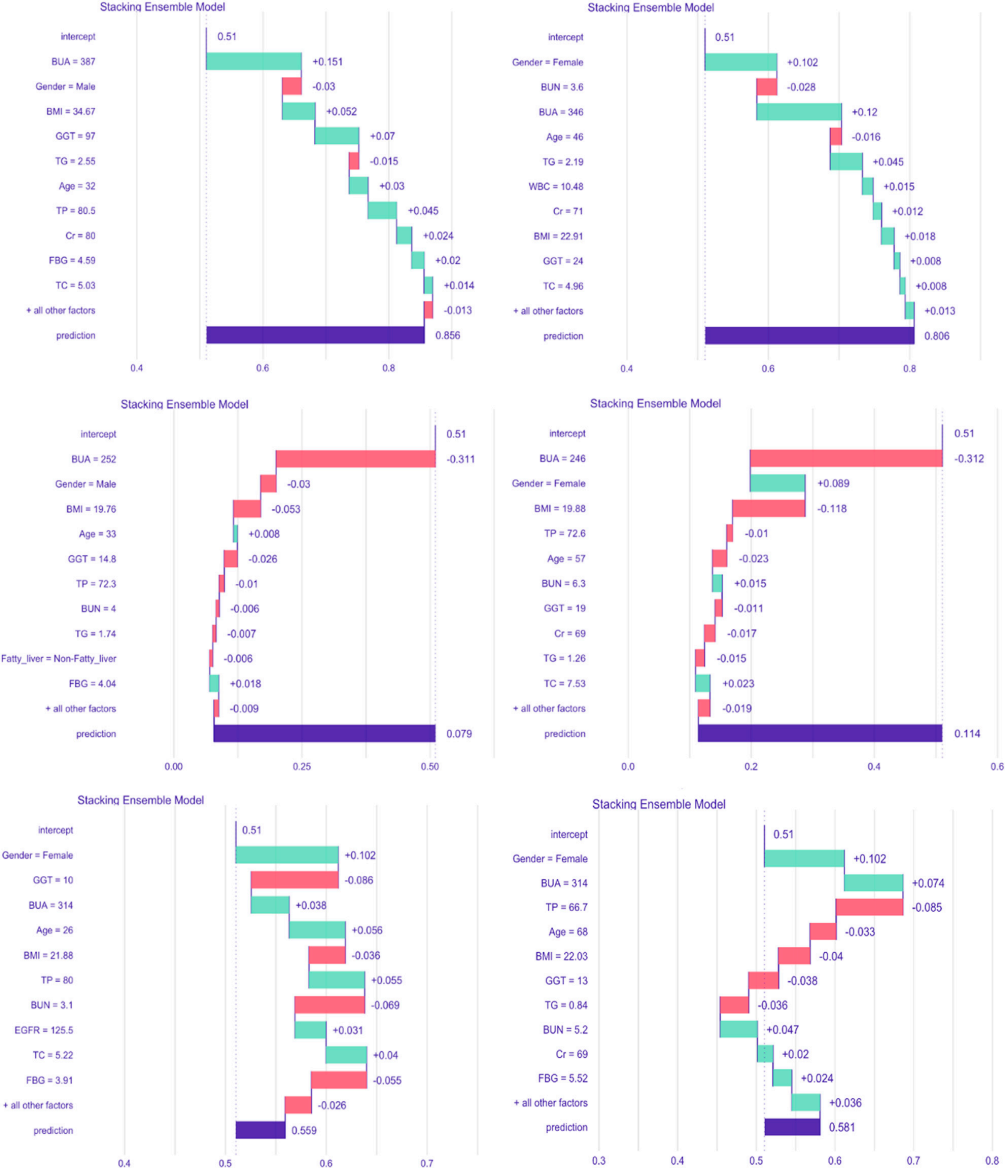
Break-down plot showing feature contributions for the stacking ensemble model. Abbreviations: BMI, body mass index; GGT, γ-glutamyl transpeptidase; TP, total protein; BUN, blood urea nitrogen; Cr, creatinine; EGFR, estimated glomerular filtration rate; TG, triglycerides; TC, total cholesterol; FBG, fasting blood glucose; WBC, white blood cell count; BUA, baseline uric acid.

To better illustrate our stacking ensemble model, the iBreakdown algorithm was used for detecting interactions for subject-level explanations. The contributing features of developing HUA in the future were estimated using six randomly selected subjects, which showed that BUA, gender, age, GGT, EFGR, BMI, TP, TG, Cr were associated with an increased risk of developing HUA. Being Female and relatively younger, together with having higher BUA, BMI, GGT, TP, TG, Cr, FBG values can increase the risk of developing HUA, as shown in Figure 6.

### Extra validation of the ensemble model

**FIGURE 7:**
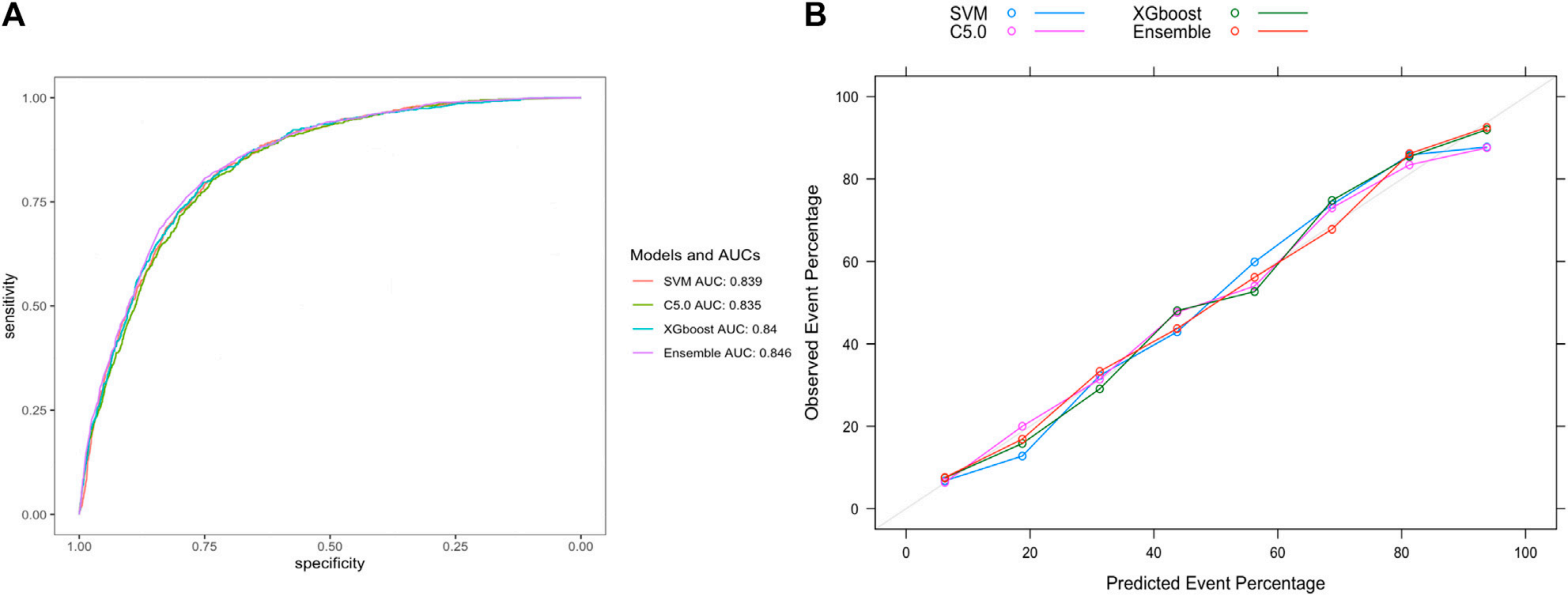
Evaluation of model performance in the extra-validation set. **(A)** ROC curve determines which model has better classification ability. **(B)** Calibration curve shows the consistency between observed and predicted probabilities. Abbreviations: ROC, the receiver operating characteristic curve; AUC, the area under the receiver operating characteristic curve; XGBoost, eXtreme gradient boosting; C5.0, decision tree C5.0; SVM, support vector machine; Ensemble, stacking ensemble model.

To further validate our model’s applicability in the health checkup population, we used another cohort from a different timespan enrolled from 1 Jan 2022, to 31 May 2023 in the same hospital, whose baseline characteristics were shown in Table 3. At the end of the follow-up period for 8559 subjects, 804 incident HUA cases were diagnosed, resulting in an incidence rate of 93.94/1000 person-years. The stacking ensemble model with an AUC of 0.846, outperformed the other three models, SVM, C5.0, and the XGBoost with AUCs of 0.839, 0.835 and 0.840, respectively, as shown in Figure 7A. The calibration curves and other metrics were also depicted, which showed our ensemble model had favorable performances in those evaluations, as shown in Figure 7B and Table 4.

**TABLE 3:** Baseline characteristics of the extra-validation set in different groups.

	Non-hyperuricemia (N = 7755)	Hyperuricemia (N = 804)	*p*-value
**Categorical variables**			
**Gender**			
Female	3529 (45.5%)	288 (35.8%)	<0.001
Male	4226 (54.5%)	516 (64.2%)	
**Fatty_liver**			
Non-Fatty_liver	4489 (57.9%)	263 (32.7%)	<0.001
Fatty_liver	3266 (42.1%)	541 (67.3%)	
**Continuous variables**			
**Age**	50.175 (14.253)	48.384 (14.333)	0.001
**BMI**	24.401 (3.374)	26.303 (3.666)	<0.001
**SBP**	128.779 (18.238)	132.158 (17.312)	<0.001
**DBP**	77.899 (11.503)	80.782 (11.608)	<0.001
**ALT**	18.682 (16.167)	24.969 (32.575)	<0.001
**AST**	18.742 (8.862)	21.508 (20.980)	<0.001
**GGT**	24.256 (25.575)	34.919 (54.285)	<0.001
**TBil**	12.214 (5.603)	12.606 (5.522)	0.034
**TP**	72.648 (3.994)	73.678 (3.830)	<0.001
**Alb**	46.603 (2.561)	47.252 (2.495)	<0.001
**BUN**	4.787 (1.244)	5.120 (1.261)	<0.001
**Cr**	73.221 (14.943)	78.112 (14.194)	<0.001
**EGFR**	102.212 (15.903)	99.045 (15.547)	<0.001
**TG**	1.298 (0.782)	1.709 (0.989)	<0.001
**TC**	4.828 (0.927)	5.063 (0.984)	<0.001
**HDL**	1.351 (0.293)	1.251 (0.277)	<0.001
**LDL**	2.874 (0.760)	3.084 (0.840)	<0.001
**FBG**	5.239 (1.343)	5.224 (1.125)	0.735
**WBC**	6.201 (1.536)	6.645 (1.543)	<0.001
**NEUT**	3.392 (1.160)	3.615 (1.146)	<0.001
**BUA**	299.381 (65.336)	362.368 (56.383)	<0.001

Note: The names of all the variable are shown in bold.

Abbreviations: BMI, body mass index; SBP, systolic blood pressure; DBP, diastolic blood pressure; ALT, alanine aminotransferase; AST, aspartate aminotransferase; GGT, γ-glutamyl transpeptidase; TBil, total bilirubin; TP, total protein; Alb, albumin; BUN, blood urea nitrogen; Cr, creatinine; EGFR, estimated glomerular filtration rate; TG, triglycerides; TC, total cholesterol; HDL, high-density lipoprotein cholesterol; LDL, low-density lipoprotein cholesterol; FBG, fasting blood glucose; WBC, white blood cell count; NEUT, neutrophil count; BUA, baseline uric acid.

**TABLE 4:** Other performance metrics of different models on the extra validation set.

Model	Accuracy	Sensitivity	Specificity	Positive predictive value	Negative predictive value	F1 score
support vector machine **(SVM)**	0.906	0.876	0.909	0.500	0.986	0.636
decision tree C5.0 (**C5.0)**	0.910	0.882	**0.912**	0.510	0.987	0.647
eXtreme gradient boosting **(XGBoost)**	0.900	0.871	0.903	0.481	0.985	0.620
stacking ensemblemodel **(Ensemble)**	**0.910**	**0.896**	0.911	**0.511**	**0.988**	**0.650**

Note: The values of the best performing models for each metric are shown in bold.

### Clinical use of the ensemble model

**FIGURE 8:**
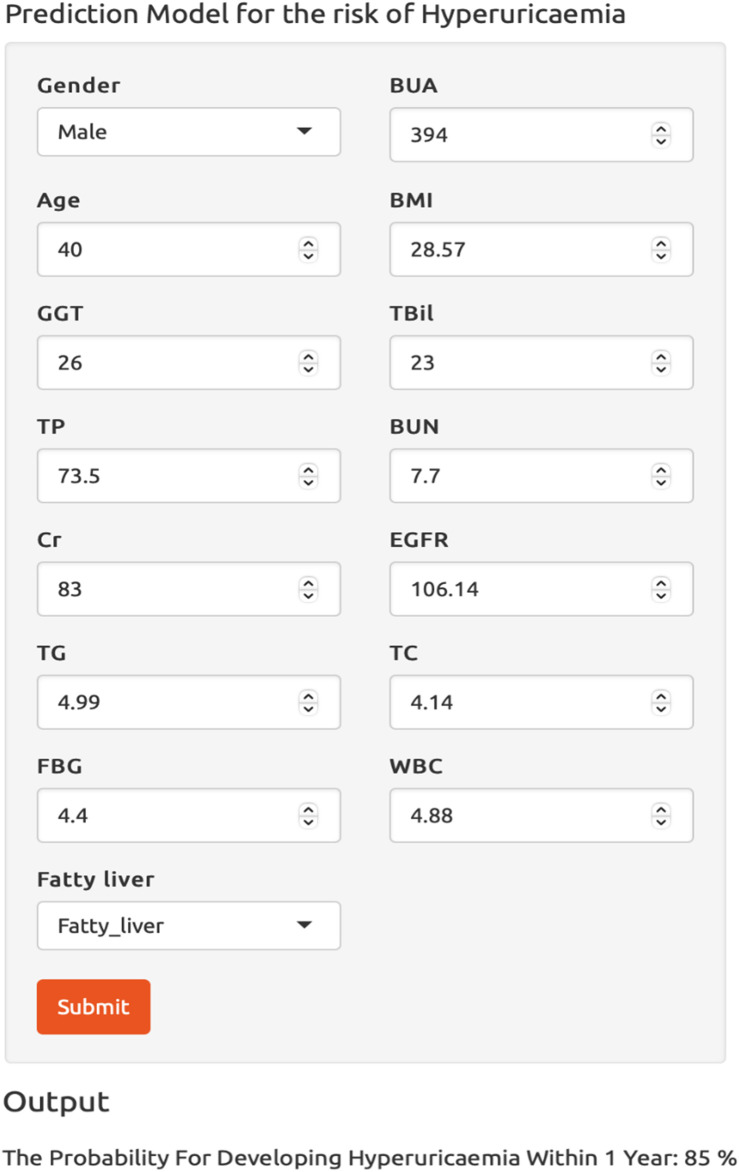
The dynamic risk calculator for hyperuricemia. Abbreviations: BMI, body mass index; GGT, γ-glutamyl transpeptidase; TBil, total bilirubin; TP, total protein; BUN, blood urea nitrogen; Cr, creatinine; EGFR, estimated glomerular filtration rate; TG, triglycerides; TC, total cholesterol; FBG, fasting blood glucose; WBC, white blood cell count; BUA, baseline uric acid.

**FIGURE 9:**
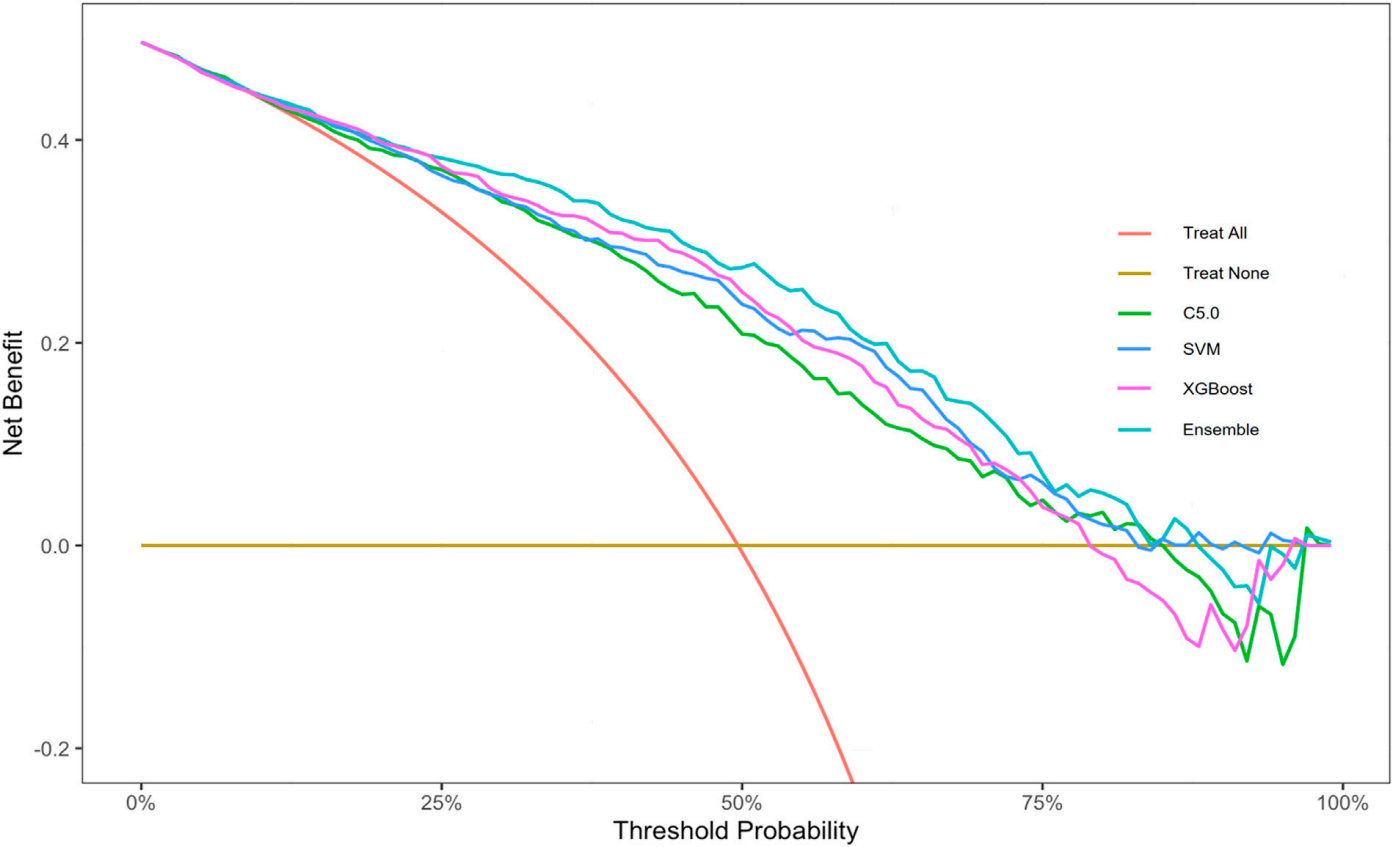
Decision curve analysis graph of the dynamic risk calculator. Abbreviations: XGBoost, eXtreme gradient boosting; C5.0, decision tree C5.0; SVM, support vector machine; Ensemble, stacking ensemble model.

To facilitate the use of our ensemble model in clinical practice, we built a dynamic risk calculator for HUA, as shown in Figure 8. To use the dynamic calculator, select or type in the correct values in the corresponding options, and click “Submit” to get the probability of developing HUA in the future. To further support our calculator’s worth, the threshold probability was analyzed using decision curve analysis, which found the minimum probability of disease at which further intervention would be warranted. As we can see from the decision curve that using the calculator based on the ensemble model to predict the risk of HUA can be clinically beneficial if the threshold ranging from around 10%–80% and more advantageous than the other three models, as shown in Figure 9.

## Discussion

In this study, a stacking ensemble prediction model for the risk of HUA was developed using data obtained from a prospective health checkup population. Our ensemble model was built up on 15 features selected by LASSO regression and demonstrated favorable performance with AUCs of 0.854 and 0.846 in the validation and extra-validation sets respectively, which outperformed the SVM, C5.0, XGBoost models. Other metrics, including accuracy, specificity, NPV, F1 score, and calibration accuracy, likewise indicated the superiority of our ensemble model and made it a powerful tool in HUA predicting.

Machine learning gives computers the ability to develop human-like learning capabilities, which allows them to solve medical problems, such as medical diagnosis, image recognition, and disease risk prediction, etc. Li et al. developed an accurate and non-invasive diagnostic model for tuberculous pleural effusion, and Fei et al. contributed to the field by creating a diagnostic model for brain diseases, showcasing the effectiveness of advanced machine learning methodologies (Li et al., 2018; Fei et al., 2020). To optimize the performance of disease classification, Xia and Houssein et al. introduced two optimization techniques, further enhancing the precision and reliability of the diagnostic models (Xia et al., 2022; Houssein and Sayed, 2023). Zhao et al. dedicated the development of accurate brain magnetic resonance images segmentation, while Emam et al. focused on refining retinal vessel segmentation algorithms (Zhao et al., 2020; Emam et al., 2023). Wei et al. constructed a useable machine learning model to predict the risk of acute kidney injury in acute respiratory distress syndrome patients (Wei et al., 2023). These breakthroughs made significant progress in solving medical problems, contributing to the improvement of diagnostic tools and techniques.

Ensemble learning is a machine learning approach that attempts to improve prediction performance by combining several weak learners into one powerful learner, which aims to reduce prediction generalization errors (Harangi, 2018; Hera et al., 2022; Zaini and Awang, 2023). Verma et al. built six different machine learning models and then developed an ensemble model using stacking and improved the performance of skin disease prediction with a final accuracy of 99.67% (Verma et al., 2020). Abdollahi and Nouri-Moghaddam used the stacking ensemble method to predict diabetes and achieved a 98.8% accuracy in disease diagnosis (Abdollahi and Nouri-Moghaddam, 2022). Our ensemble model outperformed the existing HUA prediction models in discrimination and calibration. Lee et al. explored multiple machine learning algorithms to predict HUA status in Korean individuals over the age of 40, and the random forests model performed the best with an AUC of 0.775 (Lee et al., 2019). Zeng et al. developed an artificial neural network prediction model incorporating dietary factors in Chinese adults achieving an AUC of 0.814 (Zeng et al., 2020). Gao et al. developed two different HUA random forest prediction model for male and female based on a Chinese health checkup population, and achieved AUCs of 0.730 and 0.815, respectively (Gao et al., 2021). Huang et al. developed a logistic regression prediction model for diabetic kidney disease patients based on a retrospective study achieving a C-index of 0.761 (Huang et al., 2022). Zhu et al. established a XGBoost algorithm to make an early detection of HUA risk in people taking low-dose aspirin achieving an AUC of 0.811 (Zhu et al., 2023). All these proved the advantages of the stacking ensemble strategy.

Our findings are consistent with the risk factors of HUA found in established studies. Six randomly selected subjects were analyzed using iBreakdown algorithm, which found that BUA, gender, age, GGT, EFGR, BMI, TP, TG, and Cr were associated with an increased risk of HUA. Cao and Piao both confirmed age and gender were very important factors in the development of HUA (Cao et al., 2017; Piao et al., 2022). Age is a complex influencing factor because the amount of uric acid produced varies with age. In our study, we found being relatively younger can increase the risk of developing HUA. The abovementioned two studies also proved that uric acid levels of males and females reached their apex in their 20s or so, and then declined with aging. Relatively younger people tend to have higher physical activity intensities and higher metabolic levels with different dietary habits from elderly people, which might promote them to produce more uric acid that increases the risk of developing HUA. We also found being female can increase the risk of developing HUA, which might contradict the common sense. Considering different diagnostic criteria of HUA for different genders, a female with relatively low levels of uric acid may be diagnosed with HUA, while a male must have very high levels of uric acid that could be diagnosed with HUA, two different models designed for male and female separately might be a good solution. Several other studies conducted in different countries had demonstrated significant associations between HUA and BMI, TP, and TG levels (Wang et al., 2022; Ding et al., 2023; Lyu et al., 2023). Other studies had proven smoking, drinking, sedentary lifestyle that our study did not involve could contribute to the development of HUA (Kim et al., 2018; He et al., 2022; Teramura et al., 2023). Besides these indicators studied in previous studies, we found that having relatively higher GGT and FBG values can increase the risk of HUA.

Our study has several advantages. Firstly, this cohort study included a large sample size of the cohort, which can minimize the risk of bias. Secondly, the stacking ensemble strategy was employed, which brought high predicting performance with fair robustness. Thirdly, we developed a dynamic risk calculator to predict the risk of HUA. The calculator was clear and intuitive, which could be used to quickly and accurately identify individuals at high risk of HUA. Our study has several limitations at the same time. Firstly, our results were all based on one-time measurement, which may not reflect the status of the subjects accurately and may be overestimating the incidence rate of HUA. Secondly, our HUA risk prediction model was extra-validated using datasets from the same hospital in a different timespan, while the validation data from other places were necessary. Thirdly, more variables like smoking, drinking, and dietary habits, etc. need to be explored in our analysis.

## Conclusion

Our current research has developed an accurate prediction model for the risk of HUA using a stacking ensemble technique, which has the potential to be clinically useable. The most contributing risk factors associated with HUA was also identified. This ensemble model could help in identifying high-risk HUA groups and encouraging them to pay attention to those risk factors and their unhealthy lifestyles. Although other variables like dietary habits are important factors for HUA, prediction models constructed solely from health checkup variables can be more convenient in clinical setting. In the future, we will try to include indicators for dietary habits and use external datasets to further explore our research.

## Data Availability

The raw data supporting the conclusion of this article will be made available by the authors, without undue reservation.
